# Behavioral effect of mismatch negativity neurofeedback on foreign language learning

**DOI:** 10.1371/journal.pone.0254771

**Published:** 2021-07-20

**Authors:** Ming Chang, Hideyuki Ando, Taro Maeda, Yasushi Naruse

**Affiliations:** 1 Center for Information and Neural Networks (CiNet), National Institute of Information and Communications Technology and Osaka University, Iwaoka, Iwaoka-cho, Nishi-ku, Kobe, Hyougo, Japan; 2 Graduate School of Information Science and Technology, Osaka University, Yamadaoka, Suita, Osaka, Japan; Universita degli Studi di Milano-Bicocca, ITALY

## Abstract

Listening is critical for foreign language learning. Listening difficulties can occur because of an inability to perceive or recognize sounds while listening to speech, whereas successful listening can boost understanding and improve speaking when learning a foreign language. Previous studies in our laboratory revealed that EEG-neurofeedback (NF) using mismatch negativity event-related brain potential successfully induced unconscious learning in terms of auditory discrimination of speech sounds. Here, we conducted a feasibility study with a small participant group (NF group and control group; six participants each) to examine the practical effects of mismatch negativity NF for improving the perception of speech sounds in a foreign language. Native Japanese speakers completed a task in which they learned to perceive and recognize spoken English words containing the consonants “l” or “r”. Participants received neurofeedback training while not explicitly attending to auditory stimuli. The results revealed that NF training significantly improved the proportion of correct in discrimination and recognition trials, even though the training time for each word pair was reduced to 20% of the training time reported in our previous study. The learning effect was not affected by training with three pairs of words with different vowels. The current results indicate that NF resulted in long-term learning that persisted for at least 2 months.

## Introduction

In a progressively globalized world, foreign language abilities are increasingly important. Listening is typically the basis of foreign language learning, and can be difficult for learners [1]. When speaking with another person, a learner must speak and understand the language for successful communication, and sounds must be heard before they can be reproduced. During everyday communication, adults typically spend approximately 40%–50% of their time listening, 25%–30% speaking, 11%–16% reading, and only 9% writing [2]. Furthermore, listening provides the foundation for the acquisition of language information required for speaking, reading, and writing [3]. Speech sounds that are difficult to discern in language learning tend to be difficult for learners to pronounce, read, or write [4]. Although listening is difficult for many learners, it is a critical part of the foreign language learning process, and a high level of listening ability is required for effective learning.

Difficulties in language learning may occur in the listening comprehension stage or in the perception/cognition stage that occurs before listening comprehension. These difficulties may occur because of an inability to perceive or recognize sounds while listening to speech, rather than insufficient understanding of vocabulary and grammar [5, 6]. It is possible that differences in speech sound recognition ability affect listening ability. A previous study reported that listening ability could be divided into listening comprehension (understanding of vocabulary and grammar) and speech perception (auditory perception and recognition), with speech perception occurring before listening comprehension [7]. Perception involves the recognition of a sound as belonging to a phonological category while processing auditory input. Recent studies have emphasized the significance of accurate perception of speech sounds for understanding spoken language [8, 9]. In addition, it has been reported that phoneme perception and recognition are critical for foreign language learning [10]. Theories of cross-language speech perception state that the perceived relationship between phonetic segments encountered in a foreign language plays a key role in the discrimination of those segments [11, 12]. When two sounds in a foreign language are identified as belonging to different native categories, learners can usually discriminate the two with relative ease. In contrast, discrimination may be difficult if the two sounds belong to the same native category [13, 14]. For example, native Japanese speakers are usually unable to perceive the difference between the /l/ and /r/ sounds in English [15–18]. This is well known, and is often attributed to the perceptual assimilation of /l/ and /r/ in English by a single sound, /r/, in Japanese. The Speech Learning Model (SLM) predicts that if foreign language learning begins earlier in life, learners will find it easier to establish new phonetic categories [19]. The language-specific memory traces and categories that are developed for one’s native language during early childhood [20] aid in the perception and discrimination of native speech sounds [21]. When studying a language in adulthood, non-native speech sound perception is influenced by these native categories.

Furthermore, to accurately produce a sound, it has been proposed that one must be able to accurately distinguish that same sound in reception, via what is known as the perception-production link [22]. In strong support of the existence of this link, the SLM also predicts that learners only readily create new phonetic categories when a second language sound is sufficiently different from an existing first language category [23]. Without a unique category that is used to perceive the second language sound, production of this sound is not considered to be possible [19]. Thus, to achieve proficiency in a foreign language, it is necessary to learn to discriminate fine acoustic details, such as vowels, consonants, and tones.

Acquiring fluent command of a language requires plastic changes in the neuronal circuitry of the brain that enable the correct perception of new speech sounds [24–26]. Several previous studies have reported that speech-sound representations can be examined using a cortical response called mismatch negativity (MMN) [27, 28], providing an objective index of the bottom-up processing of auditory stimulus events.

MMN is generated by the brain’s automatic response to any change in auditory stimulation that exceeds a certain limit roughly corresponding to the behavioral discrimination threshold [27]. The MMN usually peaks at 100–250 ms from the onset of the change [29], and it is elicited by a stimulus that violates a representation of the repetitive aspects of auditory stimulation. The majority of MMN studies have used simple paradigms in which frequent (standard) and infrequent (deviant) stimuli are presented in a random order, with the infrequent sound eliciting an MMN [30–32]. The MMN response is seen as a negative displacement at the frontocentral and central scalp electrodes (relative to a mastoid reference electrode) in the difference wave obtained by subtracting the event related potential (ERP) elicited by standard stimuli from that elicited by deviant stimuli. MMN is elicited without the listener attending to the sound stimulus [33]. When auditory discrimination ability is improved through behavioral training, the MMN response becomes stronger [34–36]. Therefore, the MMN component can be used as an index of the outcome of pre-attentive auditory processing. Recent studies in our laboratory [37, 38] revealed that NF based on the MMN component enabled participants to unconsciously achieve a significant improvement in the auditory discrimination of pure tones that could not previously be discriminated.

In NF [39], measurements of brain activity patterns are displayed as feedback, typically in the auditory or visual modality, and users can modulate these by adjusting their brain activity. By modulating or stabilizing the feedback signal, the user learns to regulate a particular cognitive or mental state. NF has several different modes, including that based on EEG [40–42] and on based on functional magnetic resonance imaging (fMRI) [43, 44]. fMRI-based NF can be used to measure activity in a target region of a participant’s brain in what is experienced as real time. A recent study [45] indicated that decoded fMRI-based NF without stimulus presentation led to improved visual perception. However, the decoded fMRI method requires participants to discriminate a target in advance for subsequent determination of targeted activity patterns, and thus cannot be used to train discrimination of speech sounds in a foreign language.

It may be possible to address this issue using MMN because it has been widely used and is regarded as an index of sound discrimination accuracy for discriminable auditory changes [46, 47] that occur in the absence of conscious detection [19]. Many EEG-based NF studies have used the spectral characteristics of EEG in the frequency domain, such as the alpha, theta, and other frequency bands [48–50]. Using EEG-based NF, healthy participants have been able to successfully improve their cognition and behavior by modulating their own brain activity to voluntarily increase specific EEG frequency bands [51]. In addition, MMN can be detected without identification of the brain regions involved or of the specific brain patterns associated with low-level processes.

A recent study in our laboratory revealed that the discrimination of speech sounds could be improved using MMN NF [52]. In the study [52], we conducted a training program in which native Japanese speakers learned to distinguish between the English words “light” and “right”. The program was conducted across 5 days with 12 sessions (taking approximately 1 hour each) per day. We found that in addition to improving discrimination for the presented words, MMN NF training improved the recognition of other words with the consonants “l” or “r” and a vowel in common, even though these words had not been presented during training. However, we found no improvements in performance for words with the consonants “l” or “r” that did not have a common vowel. Furthermore, the results suggested that additional training time is needed for recognition of such words without a common vowel. The training duration will be very long if training is completed for all of the different vowels in this way. Thus, a reduction in the training duration is a valuable consideration for practical applications.

Several factors underlying this training effect remain unclear. First, whether or not words that do not have a common vowel in terms of the training words used in the previous study [52] can be trained using MMN NF has not been verified. Second, the required training duration is currently unclear, and whether the training efficacy is higher if multiple words are presented in parallel training sessions or one by one in sequence has not yet been verified. Third, when training with multiple words in a single day, it is not clear whether the presentation of one word can affect performance for the other words. Finally, although changes in neural activation have been observed, whether the training has a long-term learning effect is unclear.

We addressed these issues in the present study, with the goal of improving the practicality of the training method. We hypothesized that MMN NF would produce a strong and sustained learning effect even if the training time is reduced, and MMN NF training would simultaneously improve the perception of words with different vowels. Here, we carried out the following extensions of our previous study [52]. (1) Increase in the number of training words. In our previous study, in which each participant underwent 12 training sessions per day, only one word pair was trained. In the present study, we designed the experiment to train three pairs of words with the same consonant (“l” or “r”) but different vowels. The goal was to test the efficiency of the proposed training method for a situation in which the participants learned three pairs of words in the same session. (2) Decrease in training time. In the previous study, the participants were trained for 5 days. In the present study, the participants were trained for only 3 days, with four training sessions per day. Thus, there were 12 training sessions for all pairs of words. As a result, the training duration for speech sounds was only 20% of that in our previous study. (3) Addition of follow-up test 2 months later. Although the persistence of the learning effect is very important for evaluating perceptual learning and language learning techniques, this was not verified in our previous study. Thus, in the present study, we tested whether the effects of NF training were still present 2 months after the sessions.

## Methods

**Fig 1:**
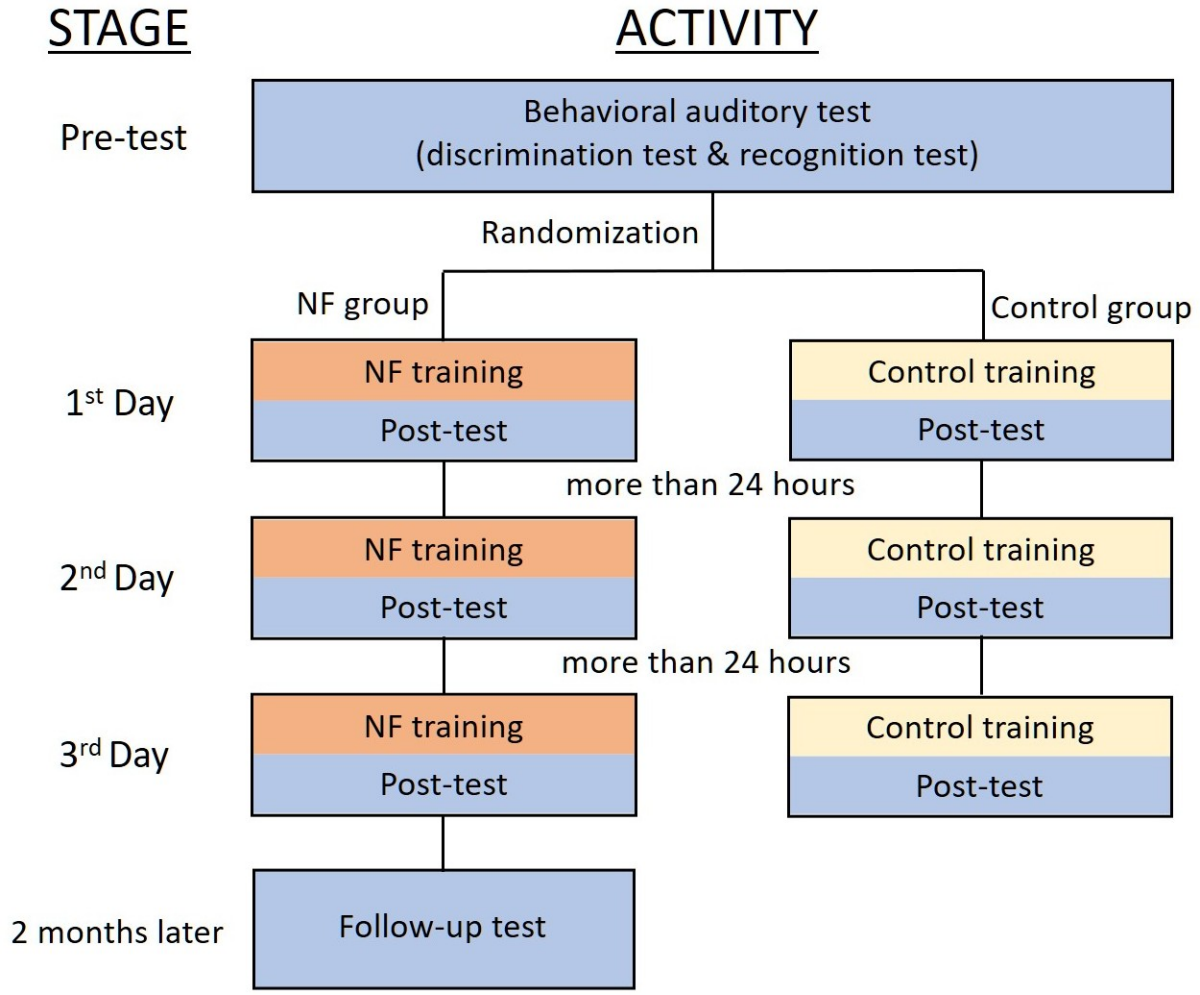
Overall study procedure.

The experimental procedure consisted of a pre-test phase, a training phase, and a post-test phase (Fig 1). We conducted a behavioral auditory test including a discrimination test and a recognition test in the pre-test and post-test phases. In the training phase, each participant learned three pairs of word sounds (see the Stimuli section below for details) on 3 different days. The training was completed within 5 days with intervals of more than 24 hours between each session. Post-tests were conducted after training on each training day. In addition, the behavioral auditory test was repeated 2 months after the end of training.

### Participants

Twelve native Japanese speakers participated in the experiment (seven men; 24–37 years old). All participants were right-handed, native speakers of Japanese, and have no experience of living abroad. The recruitment of the participants was entrusted to a company. After the recruitment conditions were posted, individuals who met the study requirements applied to be participants. The participants reported that they started learning English at school in Japan at approximately 12 years old, and that most of their exposure to English had taken place in class. None of the participants had hearing or speech disorders.

The participants were randomly assigned into two groups: a NF group and a control group. The NF group consisted of four men and two women, and the control group consisted of three men and three women. The participants were not told whether they were in the NF or control group, making this a single blind study. All experimental procedures were approved by the Ethical Committee for Human and Animal Research of the National Institute of Information and Communications Technology. Additionally, all procedures were in accordance with the Helsinki Declaration of 1964 and later revisions. Informed consent was obtained from all participants prior to the start of the study.

### Stimuli

Three pairs of stimuli were used in the experiment: (1) “light” (/laɪt/) and “right” (/raɪt/), (2) “led” (/led/) and “red” (/red/), and (3) “lead” (/li:d/) and “read” (/ri:d /). These words were synthesized using original sound editing software called “TTSEditor” (National Institute of Information and Communications Technology, Tokyo, Japan) [53]. The duration of all words was controlled to be 400 ms. These words were used to elicit MMN responses during the training procedure, and were presented in the pre-test and post-test on each training day. The stimuli were presented binaurally via earphones with an intensity of 85 dB. We checked the calibration of the earphones before the experiment to ensure that the sound stimuli were within a comfortable range and that they would not cause hearing damage.

### Behavioral auditory test

**Fig 2:**
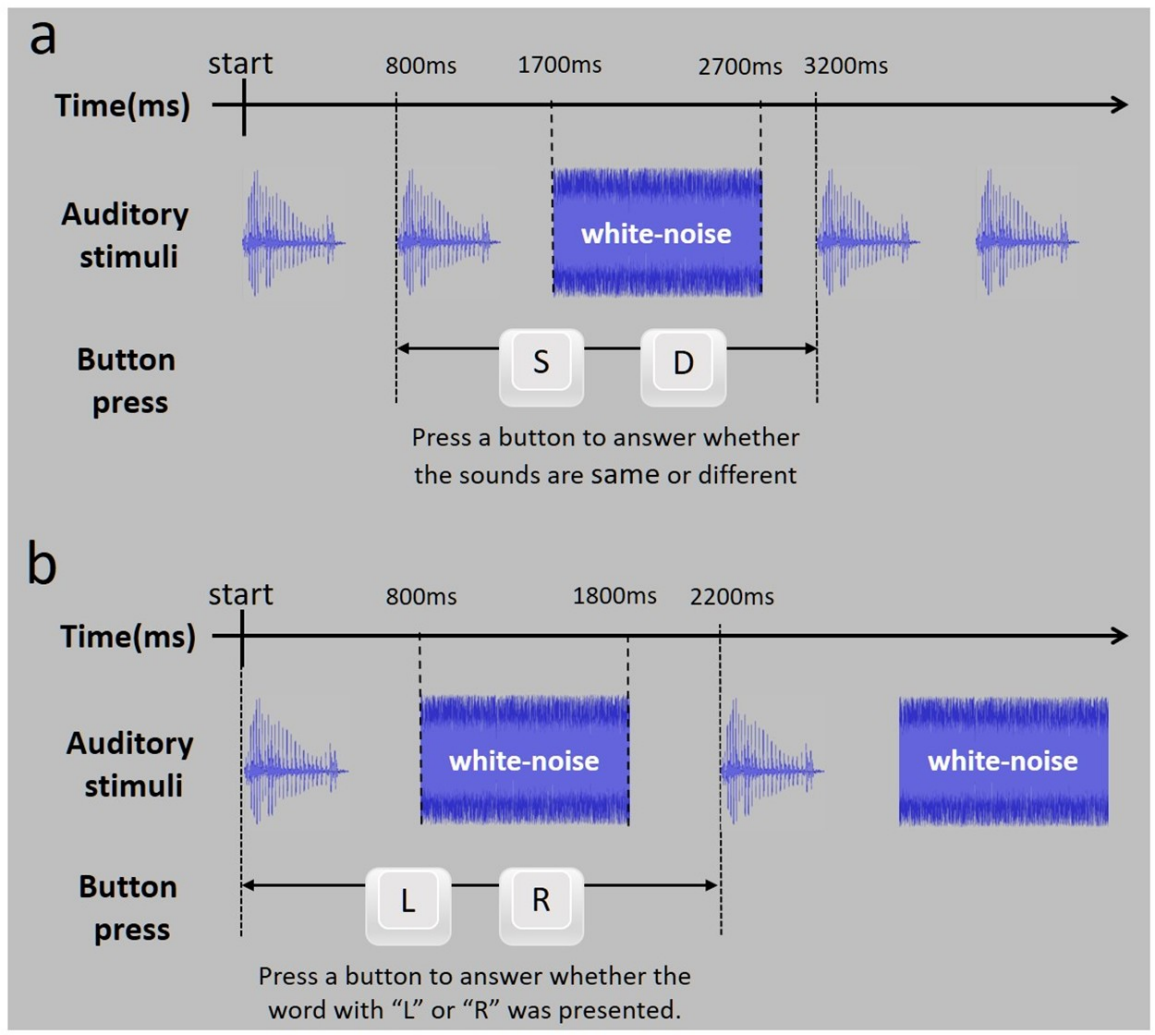
Task design of behavioral auditory tests; (a) trial sequence in discrimination task; (b) trial sequence in recognition test; *ms* milliseconds, *R* right, *L* left, *S* same, *D* different.

For the discrimination test, we used a two-alternative forced choice word pair discrimination procedure. The participants were shown word pairs with one of four combinations (e.g., for the led/red pair, the combinations were “led” and “red”, “led” and “led”, “red” and “red”, and “red” and “led”). Therefore, there were 12 combinations in total, four combinations for each of the three pairs. The order of presentation of the combinations was randomly determined and counterbalanced across trials (making eight trials for each combination). The stimulus onset asynchrony (SOA) of the two word sounds in the discrimination test was 800 ms. Throughout the task, the participants were asked to report whether the two word sounds presented in each trial were the same or different by pressing one of two buttons on a keyboard. The button presses were only registered from the presentation onset of the later word in a pair to the onset of the first word in the next pair, for a total length of 2400 ms. During each interstimulus period, 1000 ms of white noise was presented as sound interference between 500-ms silent periods that were included to reduce the effect of the previous trial on the next trial [54] (Fig 2a). The white noise was presented in every trial and was not relevant to the response made by the participant. All of the participants pressed the buttons using their right hand. There were no trials in which the participants failed to respond within the given time period.

The 96 trials in the test were divided into two blocks and performed on each experiment day. The participants were given a brief break between the two blocks of 48 trials. The participants received no feedback about the results of the trials during the experiment.

For the recognition test, we used a two-alternative forced choice task to assess behavioral auditory recognition ability. In the recognition task, the participants were presented with one word sound in each trial (randomized), and required to press the appropriate button to report which consonant (“l” or “r”) was contained in the presented word sound. The button press was registered from the start of the sound to just before the start of the next sound. During each response period, 1 s of white noise was presented as sound interference (Fig 2b). Participants pressed the buttons using their right hand. There were no trials in which the participants failed to respond within the given time period. The 96 trials were divided into two blocks and performed on 2 experimental days. Participants were given a brief break between the two blocks of 48 trials. The participants received no feedback about the results of the test.

### Electroencephalography processing and analysis

MMN was recorded in the training stage using the oddball paradigm. Words with the consonants “l” and “r” were presented as the standard and deviant stimuli in an auditory stimulus sequence, respectively. A total of 300 trials were presented (for example, “led,” 240 trials; “red,” 60 trials) in each session. On each training day, 12 sessions were presented with three word pairs in each, and each word pair was presented four times per session. The serial order of the stimuli was pseudo-random with the restriction that at least two standard stimuli were presented between deviant stimuli. The SOA between stimuli was the same as in the discrimination task. Electroencephalography (EEG) was conducted using a Wireless Biosignal Amplifier System (Polymate Mini AP108; Miyuki Giken Co., Ltd., Tokyo, Japan) and solid gel electrodes (METS INC., Chiba, Japan). In the present study, we chose measurement locations that we expected would be the most convenient for use in daily life. FPz is located near Fz and is an optimal site for easy electrode attachment because it is not covered by hair. Additionally, we previously found that it was possible to measure MMN at FPz. Therefore, we chose to record EEG at FPz, according to the international 10–20 system. In addition, electrodes were placed on the left and right mastoids as ground and reference electrodes, respectively. Electro-ocular (EOG) activity was assessed using one channel. Specifically, one electrode was placed at the upper-outer edge of the left eye to measure eye blinks and vertical eye movements. EEG activity was continuously sampled at a rate of 500 Hz, beginning 100 ms before stimulus presentation (baseline) and continuing 500 ms after stimulus onset. A band-pass filter of 0.1–35 Hz was applied online. Trials in which the EEG or EOG signal exceeded ±40 μV due to vertical eye movements and other outliers were rejected automatically. We calculated MMN amplitudes as the peak absolute values in the difference waveforms in the window 100–250 ms from the stimulus onset. Difference waveforms were obtained by subtracting the average event-related potential elicited by the standard stimuli from that elicited by the deviant stimuli.

### Training procedure

**Fig 3:**
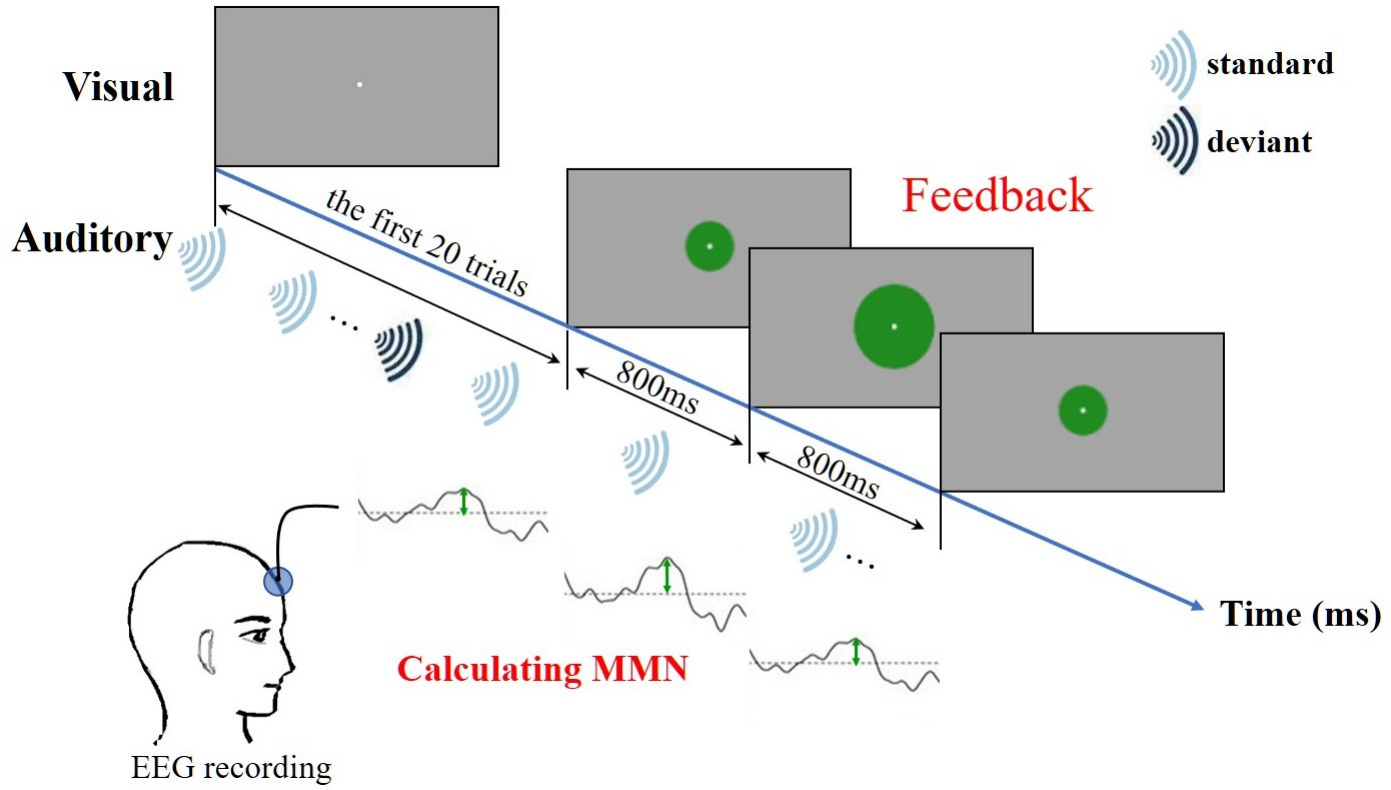
The procedure for neurofeedback training; *ms* milliseconds.

We used visual C++ 2015 to write an original program that presented the visual feedback and auditory stimuli, and recorded the EEG data. During training, the participant was seated in a comfortable chair in front of a 15.6-inch display. Sound stimuli were presented via earphones. The participant was asked to concentrate on making a solid green disc that was presented on the screen as large as possible, paying no attention to the sound stimuli. As an auditory stimulus, we presented a sequence of word sounds in a pair, in which the word sound containing the consonant “l” was the standard stimulus and the word sound containing the consonant “r” was the deviant stimulus, according to the oddball paradigm. To fix the size of the green disc, we calculated the MMN from the first 20 trials (16 standard and 4 deviant stimuli). The radius of the green disc depended on the amplitude of the MMN that was elicited by the 20 trials. From trial 21 onwards, the amplitude of the MMN was recalculated using the response from each trial instead of that on trials 1–20. Thus, the MMN was updated every 800 ms as the trials progressed (Fig 3). After the first 20 trials, the size (i.e., radius) of the disc was determined every 800 ms by linearly mapping the MMN amplitude when an MMN could be detected. The maximum possible radius of the disc was set to 4.97 degree, which corresponded with the amplitude of the MMN for 1000 and 2000 Hz tones in a preliminary experiment. In the preliminary experiment, we calculated MMN in a separate group of participants using an auditory oddball paradigm with an auditory stimulus sequence in which 1000 and 2000 Hz tones were presented as standard and deviant stimuli, respectively. As these two tones are easily distinguished from one another, we used the absolute value of the MMN amplitude elicited by 1000 and 2000 Hz tones as the maximum value (preMAX), which corresponded to the maximum possible radius of the disc (4.97 degree). Thus, even if the MMN amplitude was greater than the preMAX, the size of the disc could not exceed the maximum possible size. Based on a previous study, we measured MMN peak latencies from the most negative peak at 100–250 ms post-stimulus [29]. Therefore, only negative values were calculated. When there were no negative peaks, the value of the MMN amplitude became zero, and the size of the disc decreased to the minimum value. The size of the white fixation point was set to the minimum size of the disc (0.4 degree). The disc ranged in size from 0.4–4.97 degree, and was calculated using the following formula: SIZE = 4.57 * MMN/preMAX + 0.4 (degree). A single session consisted of 300 trials (240 standard and 60 deviant stimuli), and participants completed four sessions for each word pair. Therefore, 12 sessions (48 minutes in total) was conducted on each training day. The participants were given a break between experimental sessions.

The participants were not informed about their group assignment. The training of NF group was completed before the control group started. Although the control participants received the same stimuli and instructions as the NF group, they viewed visual feedback that was unrelated to their neural activity. Instead, the control participants received visual feedback that was randomly selected from all sessions of all participants belonging to the NF group. Thus, the control participants viewed disk sizes that corresponded to the MMN responses of the NF group, but not their own.

### Statistical analyses

To evaluate improvements in the auditory perception of the participants, we conducted a pre-test before training on the first day and post-tests after training on each training day. Behavioral data processing and statistical analyses were conducted using R-Studio (version 3.5.1). Accuracy was analyzed using a generalized linear mixed-effects model (GLMM) with the lmer function in the *lme4* package and dummy coding. Our maximal GLMM model, described in Wilkinson notation, was as follows: cbind (the number of correct responses, the total number of trials—the number of correct responses) ~ Word + Group * Day + (1 | participants). This means that the probability of correct responses was modeled using Group (control vs. NF), Day (pre vs. first day vs. second day vs. third day), and Word (light/right vs. lead/read vs. led/red) as fixed effects. We defined a participant difference as a random intercept (Participants). Moreover, to explicitly test differences between groups in terms of performance in the auditory test, we prepared a learning model fitted to a logistic function for two groups to estimate the accuracy on each day.

The neural activity data were analyzed using a two-way [Group × Day] repeated measures analysis of variance (ANOVA). Before that, we normalized the values of the MMN amplitudes based on those obtained on the first training day. We took each participant’s MMN amplitude on the first day as a baseline, and then divided the MMN amplitude for each day by this baseline. For comparison with previously reported neural activity data, we compared the training effect for the same target words “light” and “right” at the same time (12 sessions) in the NF group only. This was because these target words were used in our previous study [42]. Furthermore, in our previous study, the participants completed 12 sessions per day. In contrast, in the present study, 12 sessions were conducted over 3 days, with four sessions per day. To evaluate the effect of the spacing between the sessions, we compared the MMN amplitudes from the first four sessions and the last four sessions with those obtained in a previous study. We found that the MMN amplitudes from the first four sessions on the first training day in the previous experiment corresponded to those in all of the sessions on the first day in the present experiment. Likewise, the MMN amplitudes in the last four sessions on the first training day in the previous experiment corresponded to those in all of the sessions on the last day in the present experiment (Fig 7a). We refer to these as the first four sessions and the last four sessions in the following text. We also normalized the MMN amplitudes based on those obtained in the first session. Using the EEG data collected in the first and last four sessions, we calculated the average MMN amplitudes for all participants and submitted the data to a two-way [Experiment × Training stage] repeated measures ANOVA. Values of p < .05 were regarded as significant throughout all analyses.

Finally, on the last training day after the experiment was complete, we asked the participants how they felt that they made the disc size change.

## Results

### Improvement in behavioral auditory test performance

**Fig 4:**
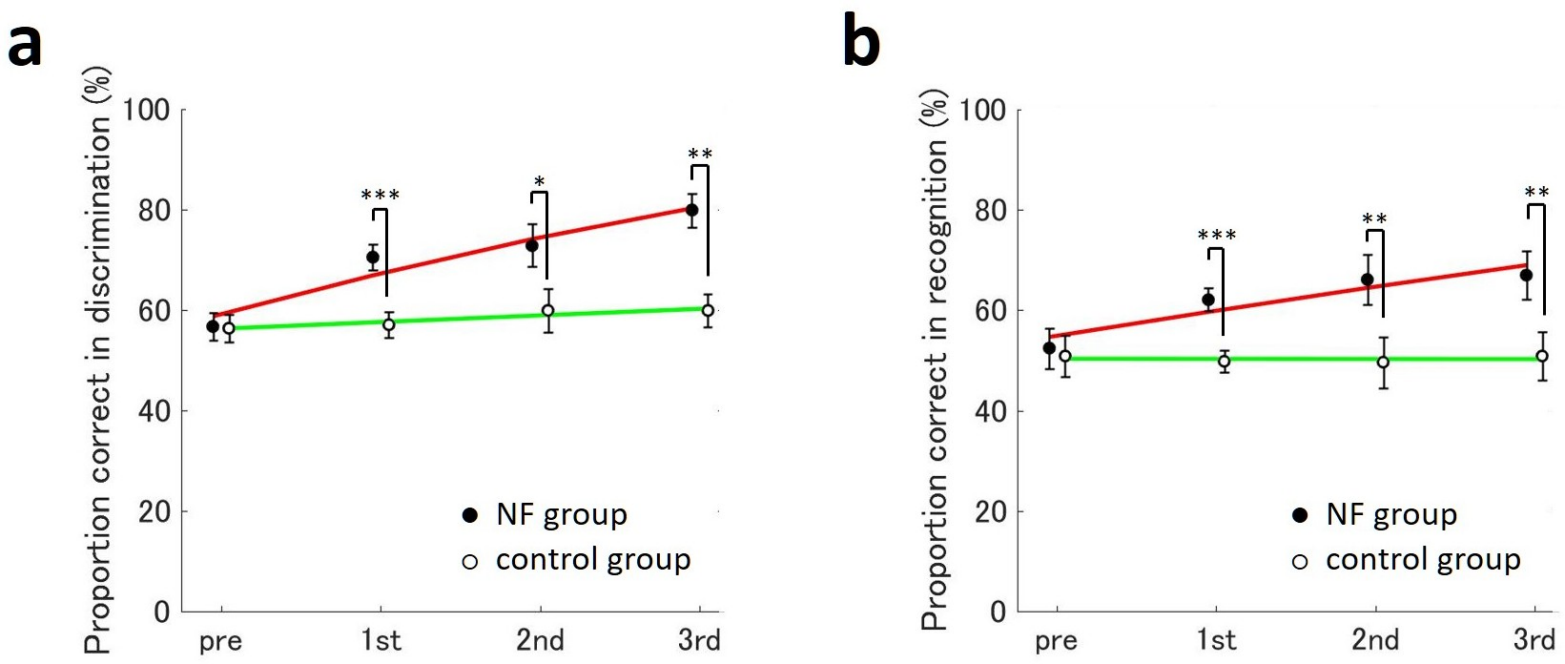
Performance in the behavioral auditory tests; (a) average proportion of correct responses and logit models for pre- and post-test data from the NF group (green line) and control group (red line) in the discrimination and (b) recognition task on each training day for the three pairs of words. Error bars represent the standard error of the mean (SEM); * p < 0.05, ** p < 0.01, *** p < 0.001.

We compared the behavioral response (the proportion of correctly discriminated and recognized words with the consonant “l” or “r”) between the pre- and post-test periods (Fig 4). The models for estimating the proportion of correct responses in the discrimination task and recognition task are summarized in Tables 1 and 2, respectively. Both models showed significant interactions between the groups and training stages. Furthermore, although Table 2 appears to show a significant difference in task performance for specific words (led/red) in the recognition task, we further analyzed the simple main effect for each word pair via multiple comparisons using Bonferroni’s correction (p = 0.114) and found no significant differences in performance between the word pairs.

**Table 1:** Proportion of correct responses in the discrimination task.

Variable	estimate	SE	z-value	p-value	
Intercept (light/right, Control, Pre)	0.25998	0.14963	1.737	0.082	
Word (lead-read)	-0.02075	0.07697	-0.270	0.788	
Word (led-red)	0.03581	0.07725	0.464	0.643	
Group (NF)	0.01105	0.20197	0.055	0.956	
Day1 (1-pre)	0.02890	0.12023	0.240	0.810	
Day2 (2-pre)	0.14582	0.12081	1.207	0.227	
Day3 (3-pre)	0.14582	0.12081	1.207	0.227	
Group (NF): Day1	0.57893	0.17356	3.336	< 0.001	***
Group (NF): Day2	0.58364	0.17525	3.330	< 0.001	***
Group (NF): Day3	0.97608	0.18092	5.395	< 0.001	***
**Variance Components**	**SD**	**Goodness of fit**			
**participants**	**0.2813**	**Log likelihood**	**-386.7**		

**Table 2:** Proportion of correct responses in the recognition task.

Variable	estimate	SE	z-value	p-value	
Intercept (light/right, Control, Pre)	0.05171	0.14529	0.356	0.722	
Word (lead-read)	0.10273	0.07453	1.378	0.168	
Word (led-red)	-0.15333	0.07403	-2.071	0.038	
Group (NF)	0.06443	0.19656	0.328	0.743	
Day1 (1-pre)	-0.04225	0.11868	-0.356	0.722	
Day2 (2-pre)	-0.05632	0.11868	-0.475	0.635	
Day3 (3-pre)	-0.04928	0.11868	-0.415	0.678	
Group (NF): Day1	0.45194	0.16977	2.662	< 0.001	**
Group (NF): Day2	0.64432	0.17089	3.770	< 0.001	***
Group (NF): Day3	0.73419	0.17165	4.277	< 0.001	***
**Variance Components**	**SD**	**Goodness of fit**			
**participants**	**0.2708**	**Log likelihood**	**-389.7**		

Fig 4a shows the proportion the correct responses and a learning model fitted to a logistic function for the two groups in the discrimination task. For the discrimination task, the model revealed no significant differences between the NF and control groups in the pre-test period (*p* = 0.919, OR = 1.01, 95%CI = 0.77–1.34). However, we found a significant difference in the average discrimination performance between the two groups on the first training day (*p* = 0.00028, OR = 1.801, 95%CI = 1.31–2.47), the second day (*p* = 0.026, OR = 1.86, 95%CI = 1.08–3.23), and the third training day (*p* = 0.0019, OR = 2.71, 95%CI = 1.44–5.08). Fig 4b shows the proportion of correct responses and a learning model fitted to a logistic function for the two groups in the recognition task. For the recognition task, the model revealed no significant differences between the NF and control groups in the pre-test period (*p* = 0.72, OR = 1.07, 95%CI = 0.75–1.52). However, we found a significant difference in the average recognition performance between the two groups on the first training day (*p* = 0.00003, OR = 1.65, 95%CI = 1.31–2.09), the second training day (*p* = 0.005, OR = 2.07, 95%CI = 1.25–3.44), and the third training day (*p* = 0.0022, OR = 2.27, 95%CI = 1.34–3.85). We observed similar results for the discrimination task and recognition task. The proportion of correct responses for each pair of words in the pre-test was not significantly different from chance (50% correct) in any group, as determined by a binomial test (the critical score for a significant difference was 57.8%).

**Fig 5:**
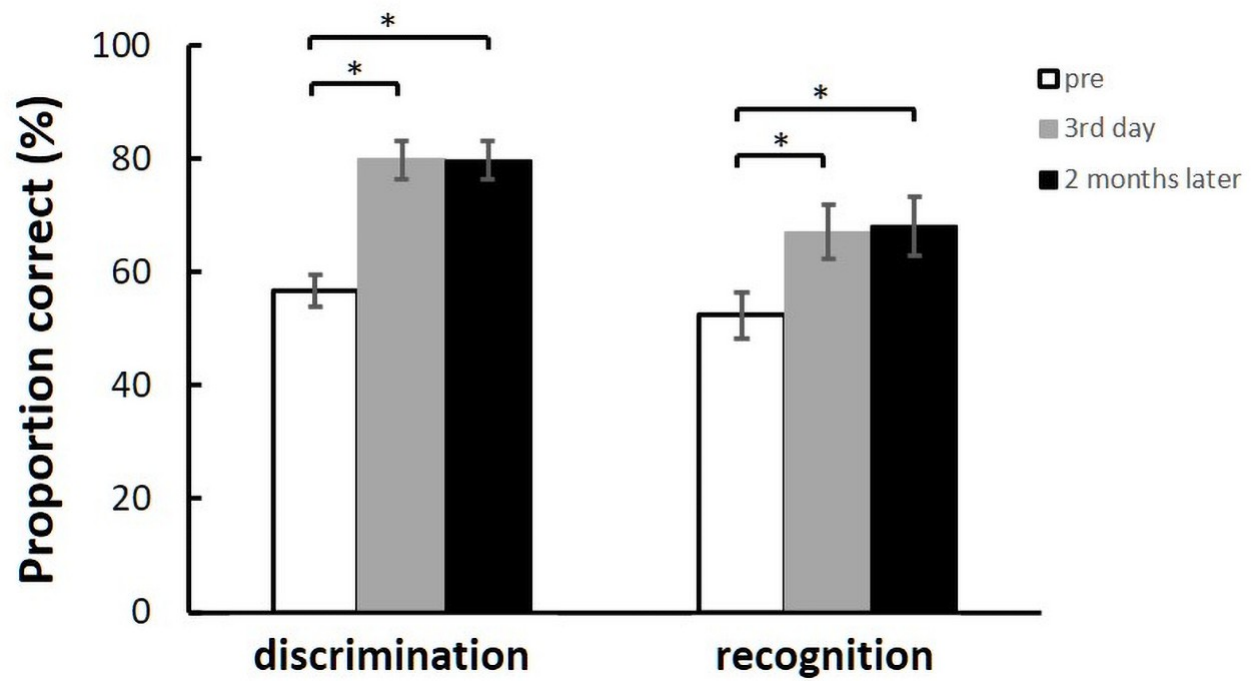
Average proportion of correct responses in terms of discrimination and recognition on the pre-test, third training day, and 2 months later in the NF group. Error bars for the data are SEM; * p < 0.05.

Because the participants in the control group did not exhibit any improvements in behavioral auditory performance after training, we thought it unlikely that they would show any improvements after 2 months without training. We performed the behavioral test 2 months after the last training day in the NF group to examine whether there was a long-term learning effect of NF training. The proportion of correct responses in each task was submitted to a one-way repeated measures ANOVA (Fig 5). For the discrimination task, the ANOVA revealed a significant effect of Test stage (F [2, 10] = 17.12, p < 0.01). The significant effect of Test stage was analyzed further for multiple comparisons using the LSD test (MSe = 61.8190, p < 0.05) to compare performance on each of the training days. The results revealed significant improvements on the post-test on both the third training day and 2 months later, compared with the pre-test performance. Similarly, for the recognition task, the ANOVA indicated a significant effect of Test stage (F [2, 10] = 5.23, p < 0.05). We further analyzed the significant effect of Test stage for multiple comparisons using the LSD test (MSe = 87.5078, p < 0.05), and compared each of the training days. The results revealed a significant improvement on both the post-test on the third training day and 2 months later, compared with the pre-test, and there were no significant differences between the post-test performance on the third training day and that on the test 2 months later.

### Improvement in neural activity

**Fig 6:**
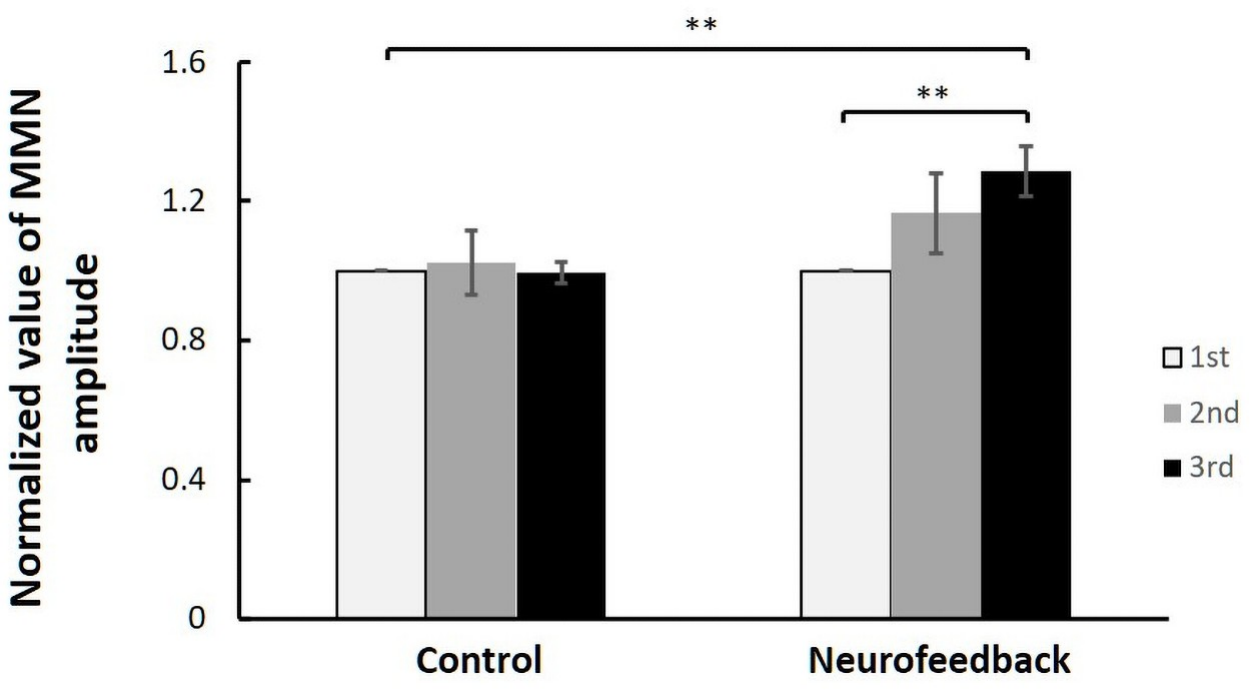
Effect of NF training on changes in neural activity. Absolute values of average MMN amplitudes for all participants on the first and third training day in the NF group and control group. Error bars for the data are SEM; ** p < 0.01.

We also assessed whether neural activity changed after training in the NF and control groups. Using the EEG data collected on the first training day and the third training day, we calculated the average MMN amplitudes for all participants and submitted the data to a two-way [Group × Day] repeated measures ANOVA. We normalized the values of the MMN amplitudes based on those obtained on the first training day. The ANOVA revealed a significant interaction between Group and Training stage (F [1, 10] = 11.88, p < 0.01). We examined the simple main effects of Group and Training stage to decompose the significant Group × Day interaction. A simple main effects test for Day revealed that the average MMN amplitudes for all participants significantly increased over time for the NF group (F [1, 10] = 22.87, p < 0.01) but not the control group (F [1, 10] = 0.01, n.s.). Thus, there was a significant improvement in performance on the third training day compared with the first training day in the NF group only (Fig 6). As shown in Fig 6, we found a significant difference in MMN amplitudes between the NF and control groups on the third training day (F [1, 10] = 11.88, p < 0.01).

### Comparison with previously reported neural activity

**Fig 7:**
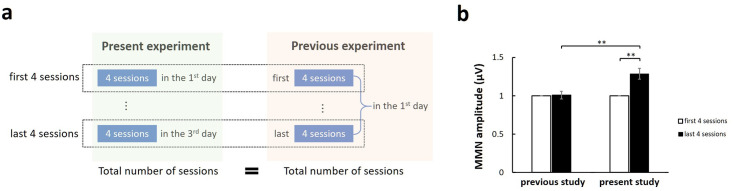
Algorithms (a) and results (b) compared with a previous study: The average MMN amplitude of the NF group for the target words “light” and “right” on the first and last four sessions in the previous and present study. Error bars for the data are SEM; ** p < 0.01.

Because we only trained the target words “light” and “right” in our previous study [52], we compared the training effects of the same target words at the same time (12 sessions) in the NF group only. As described above, the results of our previous study [52] and those of the present study indicate that significant improvements in behavioral auditory test performance occurred after NF training. Therefore, we focused on changes in neural activity. Using the EEG data collected in the first and last four sessions, we calculated the average MMN amplitudes for all participants and submitted the data to a two-way [Experiment × Training stage] repeated measures ANOVA. We normalized the values of the MMN amplitudes based on those obtained in the first sessions. The ANOVA revealed a significant interaction between Experiment and Training stage (F [1, 12] = 9.70, p < 0.01). We examined the simple main effects of Experiment and Training stage to decompose the significant Experiment × Training stage interaction. A simple main effect test for Training stage revealed that the average MMN amplitudes for all participants significantly increased over time for the NF group in the present study (F [1, 12] = 20.50, p < 0.01), but not the previous study (F [1, 12] = 0.02, n.s.). Thus, there was a significant improvement in the last four sessions compared with the first four sessions in the present study, but not the previous study (Fig 7b). As shown in Fig 7b, we found a significant difference between the present study and the previous study in terms of the MMN amplitude in the last four sessions (F [1, 12] = 9.70, p < 0.01).

On the last training day after the experiment was complete, we asked the participants how they felt that they made the disc size change. None of their responses indicated that they paid attention to the auditory stimuli during the training procedure. The participants gave explanations such as “I prayed that the green circle would enlarge”, “I tried to remember old memories or one scene in an anime”, and “I tried thinking a lot, but it did not help, so I tried not to think about anything.”

## Discussion

In the current study, to test the efficiency of the proposed NF learning method, we conducted an experiment in which participants learned to discriminate three pairs of words containing the consonants “l” and “r”, but different vowels, across 3 learning days. First, the participants exhibited significant improvements in the proportion of correct responses in terms of discrimination and recognition, even though the learning time for each pair of words was reduced to 20% of the training time in our previous study [52]. Second, the results revealed no significant differences in performance among the three pairs of words with different vowels. Thus, learning one word pair did not affect performance for the other word pairs, even though they were trained on the same day. This indicates that the learning effect was not modulated by differences in vowels. Third, we found no significant differences in performance on the behavioral test on the last training day versus 2 months later. This indicates that NF learning had a lasting effect.

In our previous study, we reported that adults were able to unconsciously learn to discriminate and recognize speech sounds in foreign languages without any behavioral training. Training was conducted across 5 days with 12 sessions (approximately 1 hour each) per day to learn one pair of target words. We found that the proportion of correct responses in the behavioral test significantly improved after training (12 sessions) on the first training day, with no significant changes in neural activity. In the current study, participants underwent 3 days of training for three pairs of word sounds, and four sessions per day were conducted for learning each pair of words. Thus, there were 12 training sessions for each pair of words, but only 20% of the training duration used in the previous study. However, we observed significant changes in behavioral performance and neural activity after the 12 sessions. Although the same number of sessions was conducted, the training in this study was completed across 3 days, leading to different results compared with the previous study [52]. The results indicate that a prolonged training period is required to change and stabilize neural activity.

Our findings indicate that it may be helpful for learners to train with multiple words in parallel, allocating relatively short training periods across several days, instead of focusing on learning only one word per day. Compared with the results of our previous study regarding actual learning effects, the current findings indicate that significant improvements occurred not only in behavioral performance, but also in neural activity, even though the time spent learning each word pair was reduced compared with our previous work. Performance on a wide variety of tasks has been found to improve when repetition of study or practice is distributed over time rather than massed, even when the total study or acquisition time is held constant [55, 56]. Referred to as the spacing effect, this has been demonstrated in domains as diverse as memory for verbal material, such as nonsense syllables, and that for words and sentences [57]. The spacing effect has been shown to have a lasting beneficial influence on long-term memory when the study phase session follows the encoding session by 24 hours. A previous study supported the importance of sleep on the long-term beneficial influence of the spacing effect [58]. The current results are consistent with these previous findings. We added a 24-h between-session break in the present experiment, and the training time was divided into three sections, which may have led to increased neural activity during the task.

The results of our previous study [52] suggested that NF training improved discrimination of consonant-vowel pairs, rather than just consonants, and that NF training produced no significant learning effect for other words containing the same consonants (“l” and “r”) but no vowel in common. Therefore, in the present study, the observed learning effect for the discrimination of /l/-/r/ sounds in one pair of words was only elicited by the NF training for this pair of words, and was unlikely to be obtained from the NF training for the other two pairs. In the domain of speech learning research, a technique called high variability phonetic training (HVPT), which is similar to our training setup, has shown great promise in increasing listeners’ ability to perceive nonnative sounds [16, 17]. In these studies [16, 17], multiple pairs of words with contrasting /l/ and /r/ sounds were also used for training. However, HVPT uses multiple voices rather than one voice, thus introducing variability into the perception practice, and the variability inherent in different voices seems to help second language learners to perceive new sounds [16, 17]. The task in the present study was designed to train multiple pairs of words with the same consonant (“l” or “r”) but different vowels in a single day, and only one synthetic voice was used. It may be possible to improve /l/-/r/ discrimination in future studies by using multiple different speakers in NF training.

Furthermore, our findings suggest that the long-term learning effects of training are the most important factor in learning. This is supported by a previous study [59] showing that behavioral performance was remarkably stable 1 month after a final training session. Another previous study [60] reported that enhanced performance in trained subjects was maintained 5–6 weeks after training. The present findings indicate that the discrimination and recognition of non-native word sounds improved following NF training, and that this learning effect persisted for at least 2 months after training. Moreover, the results indicate that neural activity was altered, and possibly stabilized, by NF training. The observed improvement in behavioral performance was rapidly acquired, and lasted for 2 months. This pattern is typically reported in skill acquisition, and has been suggested to be associated with cortical plasticity [61] reflecting training-induced, long-lasting structural changes in the cortex. Thus, long-lasting structural cortical changes may have led to the lasting effects of NF training on behavioral auditory performance in the present study. Because the participants in the control group did not exhibit any improvements in behavioral auditory performance after training, we chose not to test the control group after 2 months. However, this will be addressed in our future work. Moreover, the low number of participants (six participants per group) in the current survey limits the generalizability of the results. We plan to address this issue by increasing the participant group in our future work.

## Conclusion

The current results indicate that NF training enabled participants to learn to discriminate and recognize speech sounds in a foreign language, and that the learning effect was not modulated by differences in vowels. In addition, the current results suggest that learners are able to simultaneously learn the discrimination and recognition of /l/-/r/ contrasts in multiple words that do not have a common vowel. Based on our findings, we suggest that training for individual words should be conducted across several days, rather than focusing on learning one word each day, because it takes time to change and stabilize the neural activity underlying behavioral changes. Moreover, our results indicate that our proposed NF method has a learning effect that lasts for at least 2 months, and that auditory perceptual training is involved. Overall, we expect that such learning effects will transfer or generalize to language use/learning performance in daily life. Thus, when applying this training method in daily life, it maybe be helpful to increase the number of training words and shorten the training duration. Our NF method may represent an alternative to behavioral training for foreign language learning.

## Supporting information

S1 File (XLSX)
